# Managing schizophrenia with affective, sexual, metabolic and substance-use comorbidities: pharmacological considerations from an expert consensus

**DOI:** 10.1186/s12991-025-00620-7

**Published:** 2025-12-29

**Authors:** Eduard Vieta, Ángel L. Montejo, Toby Pillinger, Bernardo M. Dell’Osso, Maria Dimitraka, Saeed Farooq, Gustavo Jesus, Carlos Parro Torres, Thomas Wobrock, Sofia Pappa

**Affiliations:** 1 Institute of Neuroscience, University of Barcelona, Hospital Clinic, IDIBAPS, CIBERSAM, Barcelona, Catalonia Spain; 2 Institute of Biomedical Research, Hospital Universitario de Salamanca, IBSAL, Universidad de Salamanca, Salamanca, Spain; 3 Department of Psychosis Studies, Institute of Psychiatry, Psychology and Neuroscience, King’s College London, London, UK; 4 Department of Mental Health and Addictions, University of MilanASST Fatebenefratelli-Sacco, Via G.B. Grassi 74, Milan, 20157 Italy; 5 Department of Psychiatry and Behavioral Sciences, Stanford School of Medicine, Stanford University, Stanford, CA USA; 6 Multidisciplinary Mental Health Hospital of Attica, 1 st Adult Psychiatric Clinic, Athinon av 374, Athens, Greece; 7 National Institute of Health and Care Research, School of Medicine, Keele University and Midlands Partnership NHS Foundation Trust, Keele, UK; 8 Department of Psychiatry and Mental Health, Lisbon Medicine Faculty, Hospital de Vila Franca de Xira, National Health Service, Catolica Medical School, University of Lisbon, Lisbon, Portugal; 9 Institute of Psychiatry and Mental Health, Gregorio Marañón University General Hospital, Madrid, Spain; 10 Centre of Mental Health, County Hospitals Darmstadt-Dieburg, Groß-Umstadt, and Department of Psychiatry and Psychotherapy, University Medical Center Gööttingen, Göttingen, Germany; 11 Department of Brain Sciences, Faculty of Medicine, Imperial College London, West London NHS Trust, London, UK; 12 Interventional and Precision Psychiatry Service (IPPS), West London NHS Trust, London, UK

**Keywords:** Schizophrenia, Comorbidities, Antipsychotics, Pharmacological management, Real-world practice, Individualized treatment, Affective symptoms, Sexual dysfunction, Metabolic disturbances, Substance use disorders.

## Abstract

**Background:**

Schizophrenia is a complex psychiatric disorder frequently complicated by comorbidities that contribute to functional impairment, poor treatment adherence, and elevated mortality. Among the most prevalent and burdensome are affective symptoms, sexual dysfunction, metabolic disturbances, and substance use disorders, which remain underrecognized and insufficiently addressed in routine care.

**Objectives:**

This expert consensus aimed to develop comorbidity-informed pharmacological strategies for schizophrenia, grounded in real-world challenges and individualized treatment needs.

**Methods:**

A multidisciplinary panel of 10 European psychiatrists convened for an in-person meeting. Four expert-led presentations, each addressing a key comorbidity, were followed by open discussion. A modified two-round Delphi process was subsequently used to validate the consensus statements, which were thematically synthesized into actionable recommendations.

**Results:**

Consensus-based recommendations were developed across four comorbidity domains, integrating current evidence with real-world clinical expertise. The panel emphasized the importance of individualized, patient-centered pharmacological strategies that balance efficacy, tolerability, and long-term functional outcomes. Partial dopamine agonists and other metabolically or hormonally favorable agents were identified as clinically useful across several comorbid profiles. Recommendations also addressed the optimization of antipsychotic selection, management of treatment-emergent side effects, and coordinated care for patients with dual diagnosis (also referred to as co-occurring disorders). Measurement-based monitoring and integrated service models were consistently highlighted as essential for improving outcomes.

**Conclusions:**

Effective management of schizophrenia requires a shift toward comorbidity-informed, recovery-oriented pharmacological care. These expert recommendations provide practical strategies to support individualized treatment planning in real-world clinical settings.

## Introduction

Schizophrenia is a severe psychiatric disorder characterized by disruptions in thought, emotion, and behavior. It affects an estimated 0.3–0.7% of the global population, with considerable variation across regions and study methodologies, and imposes a substantial and enduring burden on patients, caregivers, and healthcare systems [1–4]. The condition is defined by marked heterogeneity in its etiology, symptomatology, and course [5]. Hallmark features include positive symptoms such as hallucinations and delusions, negative symptoms such as social withdrawal and reduced motivation, as well as cognitive impairments and affective disturbances [6–8]. These symptoms often fluctuate in severity and duration, contributing to a relapsing-remitting pattern characterized by incomplete remissions, persistent disability, and shortened life expectancy.

### Burden of comorbidities in schizophrenia

Beyond core psychotic symptoms, schizophrenia is frequently complicated by comorbid conditions that exacerbate the illness burden and hinder recovery [9, 10]. Among the most prevalent and clinically challenging are affective symptoms and disorders, sexual dysfunction, metabolic disturbances, and substance use disorders (SUDs). These comorbidities interfere with treatment adherence, worsen both psychiatric and physical outcomes, and significantly increase the risk of early mortality [11], particularly due to cardiovascular disease and suicide [12–14]. On average, individuals with schizophrenia die 15–20 years earlier than the general population, primarily from preventable physical health conditions such as cardiovascular disease, type 2 diabetes, and stroke, rather than from direct psychiatric causes [12, 14, 15]. Cardiometabolic disease remains the dominant contributor to this mortality gap, driven by a complex interplay of adverse lifestyle behaviors, systemic healthcare disparities, medication-induced side effects, and possibly intrinsic biological vulnerabilities.

### Overview of key comorbidities

Affective symptoms and disorders, including depression, anxiety, dysphoria, aggression, and psychomotor agitation, affect 40–60% of individuals with schizophrenia and are strongly associated with relapse, suicidality, and poor quality of life [1, 9, 16]. Sexual dysfunction is similarly prevalent and frequently underrecognized in schizophrenia, affecting approximately 50–56% of patients, with slightly higher rates in women than men. This may stem from multiple interacting factors. Neurobiological and psychosocial aspects of the illness itself – including negative symptoms (e.g., anhedonia, avolition) – can impair sexual desire, functioning, and intimacy. In addition, antipsychotic medications are a major contributor, particularly through hyperprolactinemia induced by antagonism of the dopaminergic tuberoinfundibular pathway. Several agents also have antihistaminergic and anticholinergic properties that may further impair sexual function. Physical comorbidities that are more common in this population, such as diabetes, obesity, and cardiovascular disease, can also negatively affect sexual health through vascular and endocrine mechanisms [17–20]. Despite this high prevalence, clinical detection remains low. A large Spanish study found that although 46% of patients experienced sexual dysfunction, only about one-third spontaneously reported symptoms to clinicians [17]. Metabolic disturbances, including obesity, insulin resistance, diabetes, and dyslipidemia, are also highly prevalent and represent major contributors to cardiovascular risk and reduced life expectancy [21, 22]. Psychotropic agents vary widely in their metabolic side-effect profiles, requiring careful selection and proactive monitoring [23–28]. Although the metabolic risks of antipsychotics are well established, other commonly prescribed psychotropic medications, such as antidepressants and mood stabilizers, can also contribute to weight gain and broader metabolic disturbances [29]. SUDs affect approximately 42% of individuals with schizophrenia, most commonly involving tobacco, alcohol, cannabis, and stimulants [30–32]. Tobacco use, in particular, is highly prevalent, affecting up to 90% of patients, and remains the leading preventable contributor to excess morbidity and mortality [33]. Comorbid SUD is associated with worsened psychiatric symptoms, reduced treatment adherence, and increased risks of hospitalization, mortality, homelessness, legal issues, and victimization [32–34]. Several explanatory models help frame the complex, bidirectional relationship between schizophrenia and SUD [35]. These include: the diathesis-stress model, which proposes that substance use may trigger illness onset in genetically predisposed individuals; the self-medication model, which views substance use as a maladaptive strategy to manage symptoms or medication side effects; the cumulative risk model, emphasizing the additive effects of genetic, neurodevelopmental, and psychosocial vulnerabilities; and the reward circuitry dysfunction model, which implicates overlapping dopamine and glutamate dysregulation in both psychosis and addiction [36, 37]. Despite their theoretical differences, these models converge on a common insight: schizophrenia and SUD mutually reinforce one another, intensifying clinical burden and complicating care, underscoring the need for early detection, integrated services, and tailored pharmacological interventions [38].

### Pharmacological context

Pharmacological treatment remains central to the management of schizophrenia, particularly for controlling positive symptoms, with dopamine D2 receptor antagonism as the historically primary therapeutic mechanism [39]. The evolution of antipsychotic treatment reflects a growing awareness of schizophrenia’s clinical heterogeneity and complexity [40, 41]. First-generation antipsychotics (e.g., chlorpromazine, haloperidol), introduced before the 1960 s, effectively reduced positive symptoms but frequently caused extrapyramidal side effects (EPS) [42], and did not improve negative and depressive symptoms [43]. Between the 1960 s and 1990 s, treatment goals expanded to include relapse prevention and clinical stability, leading to the adoption of second-generation antipsychotics [44]. Although these agents demonstrated a markedly reduced risk of EPS, they introduced new challenges, particularly an increased risk of metabolic complications. Other adverse side effects, including sedation, hyperprolactinemia, and sexual dysfunction, also remained common concerns [45–48]. Moreover, both first- and many second-generation agents show limited efficacy for negative and cognitive symptoms, which are major determinants of long-term disability [49, 50].

Since the early 2000 s, treatment goals have further evolved from symptom control to a broader emphasis on functional recovery, quality of life, and personalized care. Among the newer third-generation antipsychotics, aripiprazole, brexpiprazole, and cariprazine offer broader efficacy across symptom domains – positive, negative, affective, and cognitive – while offering a lower risk of metabolic side effects and EPS [41, 51]. Although akathisia may occur early, these agents generally demonstrate improved long-term tolerability compared with other antipsychotics [41]. Although all three are partial dopamine D2/D3 receptor agonists, they differ meaningfully in their pharmacodynamic properties [41, 51]. Aripiprazole has higher intrinsic D2 activity and a broader dopaminergic activation profile, contributing to its activating potential in some patients. Brexpiprazole has lower intrinsic D2 activity and greater engagement of 5-HT1A and α1B receptors, resulting in a more balanced and less activating profile [41]. Cariprazine, with preferential affinity for D3 receptors, may be particularly relevant in negative, cognitive, and affective symptoms, with emerging real-world evidence in SUD comorbidity [52, 53]. These distinctions underscore that partial agonists are not therapeutically interchangeable and may be differentially suited to specific comorbidity profiles. All antipsychotics referenced in this consensus are approved in at least one European Union country and are in current clinical use within Europe, ensuring real-world applicability.

### The need for comorbidity-aware, measurement-based, patient-centered care

Despite increasing recognition of the importance of comorbidities, their systematic assessment and proactive management remain inconsistently implemented in routine psychiatric care. Stigma, diagnostic overshadowing, service fragmentation, and insufficient specialized training frequently prevent individuals from receiving holistic treatment. Measurement-based care, which involves the regular use of validated tools to monitor symptoms, side effects, and functional outcomes, has the potential to improve treatment engagement, support shared decision-making, and reduce disparities. Psychiatrists must adopt a leadership role in coordinating care that addresses both mental illness and physical health (including reproductive and sexual well-being). SUDs, as mental illnesses, fall within this scope and require integrated approaches alongside other psychiatric and physical health needs [54–56].

Nevertheless, practical comorbidity-informed pharmacological guidance remains scarce in real-world settings. Randomized controlled trials, while essential for establishing drug efficacy and safety, are typically conducted under restrictive conditions that exclude patients with complex comorbid profiles [57]. As a result, their findings may not fully reflect the challenges encountered in everyday clinical care. In practice, clinicians often rely on treatment recommendations and algorithms derived from textbooks, guidelines, or expert panels. However, these may not always align with the complexities of managing schizophrenia in patients with significant comorbidity burdens [58]. Some guidelines summarize certain comorbidities and provide distinct treatment recommendations; however, studies including schizophrenia patients with comorbidities are sparse [59].

Both the Lancet Psychiatry Physical Health Commission and the INTEGRATE guidelines highlight the urgent need to embed physical health considerations into psychiatric care [26, 60]. These recommendations emphasize the early prioritization of metabolic health, particularly in first-episode psychosis. One key strategy is the preferential use of metabolically favorable antipsychotics, such as partial dopamine agonists. They also call for structured metabolic monitoring (e.g., weight, glucose, lipid levels) and stronger collaboration between psychiatry, primary care, and endocrinology as essential components of integrated care.

### Objectives and scope of the expert consensus

The objective of this expert consensus was to develop practical, comorbidity-informed pharmacological strategies for real-world settings, focusing on the four high-impact comorbidities complicating schizophrenia – affective symptoms, sexual dysfunction, metabolic disturbances, and SUDs. The recommendations aim to support clinicians in balancing symptom control with long-term tolerability, physical health, and functional recovery, emphasizing individualized, patient-centered, and comorbidity-aware treatment strategies.

## Methods

A European panel of experts was convened to explore pharmacological management strategies for schizophrenia with four high-impact psychiatric and physical comorbidities: affective symptoms, sexual dysfunction, metabolic disturbances, and SUDs. The panel included 10 clinicians with recognized expertise in psychiatry and psychopharmacology. Participants were selected based on their clinical leadership, academic contributions, and substantial real-world experience in managing schizophrenia and its associated comorbidities. Expertise was defined broadly, encompassing scientific knowledge, domain-specific leadership, and extensive clinical experience across metabolic psychiatry, sexual medicine, dual diagnosis, early psychosis, and guideline development, rather than publication metrics alone. The panel represented Germany, Greece, Italy, Portugal, Spain, and the UK, contributing perspectives from academic psychiatry, hospital and community mental health services, clinical research, and health policy.

A structured expert consensus process was used to develop the recommendations. Four subject-matter experts led discussions in their respective domains: affective symptoms (Eduard Vieta), sexual health (Ángel L. Montejo), metabolic disturbances (Toby Pillinger), and SUDs (Bernardo M. Dell’Osso). Each conducted a targeted literature review and compiled a curated set of peer-reviewed articles, clinical guidelines, and other relevant materials. These materials were shared with all panel members before the meeting to ensure consistent preparation and alignment.

The in-person meeting was held on 15 May 2025, in Florence, Italy, and lasted approximately 6 h. The meeting was moderated (Sofia Pappa) and followed a semi-structured format that prioritized open dialogue over formal scoring. Each domain expert began with a concise, evidence-based overview of their topic, highlighting major clinical challenges and pharmacological considerations. These presentations formed the basis for collective discussion, during which the panel evaluated available evidence, shared real-world experiences, and iteratively refined preliminary recommendations.

To ensure methodological rigor and independence, several safeguards were implemented. All evidence appraisal, discussion, and drafting of recommendations were undertaken exclusively by the expert panel. Although the initiative received unrestricted educational support, sponsor representatives did not participate in literature review, expert discussion, statement development, Delphi voting, or manuscript preparation. Structured and anonymous Delphi rounds were used to minimize dominance effects and support independent judgment.

Following the meeting, recommendations were validated using a two-round modified Delphi process. Experts rated each statement using a 7-point Likert scale (1 = strongly disagree; 7 = strongly agree), with the option to provide qualitative comments. Consensus was predefined as ≥ 75% of panelists rating a statement 6 or 7. The Delphi procedure followed key principles of the RAND/UCLA Appropriateness Method, a widely accepted framework for transparent, structured consensus development [61]. Consistent with this approach, the process included independent preparation of evidence summaries by domain experts, generation of draft statements during the in-person meeting, two anonymous voting rounds, a predefined consensus threshold, and avoidance of consensus as a stopping rule to prevent forced agreement. This approach aligns with contemporary European Delphi applications [62].

Statements reaching consensus in Round 1 were accepted. Based on qualitative feedback from Round 1, two additional statements were introduced and evaluated in Round 2. Final interpretations were based on percentage agreement, with ≥ 90% classified as very strong agreement, 80–89% as strong consensus, 75–79% as consensus achieved, and 60–74% as moderate agreement. These rating distributions (percentage agreement and median scores) are reported in the corresponding tables. Qualitative dissenting views were captured during both the meetings and the Delphi rounds and informed refinement of statements when appropriate. No additional quantitative data were generated beyond those presented in the Results.

Data synthesis followed a thematic approach. For each comorbidity domain, findings were organized into three sections within the Results: (1) Clinical challenges and real-world reflections, synthesizing the evidence-based presentation by the domain expert and the subsequent panel discussion; (2) Pharmacological strategies and key considerations, reflecting treatment approaches and considerations drawn from both the expert’s overview and collective panel insights; and (3) Expert consensus with actionable recommendations, developed and validated as formal statements through the modified Delphi process. Where relevant, the first two sections were supplemented with published evidence to contextualize and support the panel’s insights.

## Results

### Affective symptoms in schizophrenia

#### Affective symptoms: clinical challenges and real-world reflections

Affective symptoms are intrinsic to the course of schizophrenia, with mood disturbances that may precede, coincide with, or follow psychotic episodes. This longitudinal complexity challenges the adequacy of traditional diagnostic frameworks such as the Diagnostic and Statistical Manual of Mental Disorders (DSM) and the International Classification of Diseases (ICD). Depression, anxiety, dysphoria, aggression, and psychomotor agitation are highly prevalent and strongly associated with poor prognosis, functional impairment, and reduced quality of life [1, 9, 16]. Despite their relevance, affective symptoms have historically received less attention in both research and clinical care.

A dimensional, function-oriented approach, focused on symptom trajectory, functional impairment, and long-term outcomes, may offer greater clinical utility than rigid categorical classifications. For example, schizoaffective disorder remains controversial, while features such as family history of bipolar disorder or early affective symptoms may better predict a mood-related illness course. Therefore, effective management should be individualized and patient-centered, addressing recovery goals, functional improvement, and quality of life alongside symptom control.

Accurate diagnosis often evolves over time. Early depressive symptoms in prodromal or high-risk states require careful interpretation. Naturalistic studies [63] suggest that selective serotonin reuptake inhibitors may be beneficial in early illness phases, particularly when mood symptoms predominate and psychosocial support is available. However, trials such as PREVENT [64] emphasize the value of structured interventions such as cognitive behavioral therapy and the importance of careful risk–benefit assessment when considering antipsychotics in at-risk individuals.

Sex and hormonal factors substantially influence symptom presentation and treatment response. For instance, women may experience higher antipsychotic serum levels at standard doses, especially during menopause, increasing the risk of side effects. In such cases, dose adjustments or hormone replacement therapy may be more appropriate than antidepressants for managing mood and cognitive symptoms. Finally, long-term, unadjusted pharmacological regimens can limit optimal care, with treatments often continued despite suboptimal benefits. Periodic review and proactive treatment adjustment are recommended to align management with evolving patient needs.

#### Affective symptoms: pharmacological strategies and key considerations

Affective symptoms often emerge early in the illness and may persist despite remission of positive symptoms. As strong predictors of relapse, suicidality, and long-term disability, they require targeted pharmacological approaches that address the full spectrum of schizophrenia [65]. Antipsychotics with intrinsic antidepressant properties, such as lurasidone, quetiapine, and selected partial dopamine agonists (e.g., cariprazine), are preferred for patients with affective symptoms due to their dual efficacy in managing psychotic and mood disturbances and their generally favorable tolerability profiles.

Tolerability remains a key determinant of adherence. Side effects such as sexual dysfunction, weight gain, and extrapyramidal symptoms should be identified early and managed proactively. Antidepressants with a high sexual side-effect burden (e.g., paroxetine) may be particularly problematic. In clinical practice, antipsychotics that balance broad symptom control with a lower side-effect burden are increasingly favored [66, 67]. Adjunctive antidepressants may be cautiously considered in selected cases. However, concerns remain about their limited efficacy and potential to worsen symptoms if not used alongside antipsychotic treatment. A switch to an antipsychotic with better mood-stabilizing properties is generally preferred before initiating adjunctive antidepressants.

Mood stabilizers are appropriate in the presence of bipolar-spectrum features or clear cyclical mood symptoms, but are not recommended for schizophrenia without these characteristics. Family history and longitudinal mood patterning are key factors in guiding their use.

When monotherapy is insufficient, tailored antipsychotic combinations based on pharmacological rationale may be preferable to dose escalation, especially when agents with high D2 blockade (e.g., haloperidol, risperidone) are involved [68].

Given the lack of real-world outcome data on affective domains in schizophrenia, a network meta-analysis comparing antipsychotics by mood-related efficacy is warranted. Until such evidence becomes available, treatment decisions should remain individualized and guided by recovery-oriented goals. Partial dopamine agonists have demonstrated therapeutic benefits across symptom domains, including bipolar features, motivational deficits, and cognitive symptoms. Real-world evidence further supports the effectiveness of newer antipsychotics in everyday clinical settings, reinforcing their utility beyond controlled trials [69–71].

#### Affective symptoms: expert consensus with actionable recommendations

The panel developed eight consensus statements addressing the identification, assessment, and management of affective symptoms in schizophrenia. These recommendations emphasize the integration of evidence-based pharmacological strategies with real-world clinical priorities, supporting both psychiatric and functional recovery goals. All eight statements achieved consensus in Round 1, with five rated as very strong agreement and three rated as strong consensus (Table 1).

**Table 1:** Affective symptoms – Delphi consensus statements and level of agreement

No.	Statement	% Agree or Strongly Agree	Median Score	Interpretation
1	Affective symptoms are central to the clinical trajectory of schizophrenia and warrant greater prioritization in both clinical care and research	80%	6.5	Strong consensus
2	Pharmacological decision-making should incorporate mood symptomatology and prioritize antipsychotics with demonstrated efficacy in both psychotic and affective domains	80%	7	Strong consensus
3	Treatment plans should align with patient-defined outcomes, such as social relationships, work functioning, and overall quality of life, rather than focus solely on symptom remission	100%	7	Very strong agreement
4	Temporal patterns, cyclical mood fluctuations, and family history should inform diagnostic and treatment decisions, especially when distinguishing between schizophrenia, bipolar disorder, or schizoaffective disorder	90%	7	Very strong agreement
5	Hormonal and sex-specific variables should be systematically addressed in both treatment selection and guideline development. This includes dose adjustments during menopause and consideration of hormonal interventions	100%	7	Very strong agreement
6	A dimensional, recovery-oriented perspective is preferred over categorical diagnostic labels, allowing more flexible and personalized care	80%	7	Strong consensus
7	Regular, structured reviews of treatment are essential to avoid therapeutic inertia. Proactive follow-up and outcome monitoring should guide adjustments when current strategies fail to meet patient goals	100%	7	Very strong agreement
8	When antidepressants are considered, they should be carefully selected and monitored, avoiding routine combinations and prioritizing agents with a lower risk of cognitive, metabolic, and sexual side effects	100%	7	Very strong agreement

Box 1 summarizes pharmacological options commonly considered for managing affective symptoms in schizophrenia, based on expert insights.


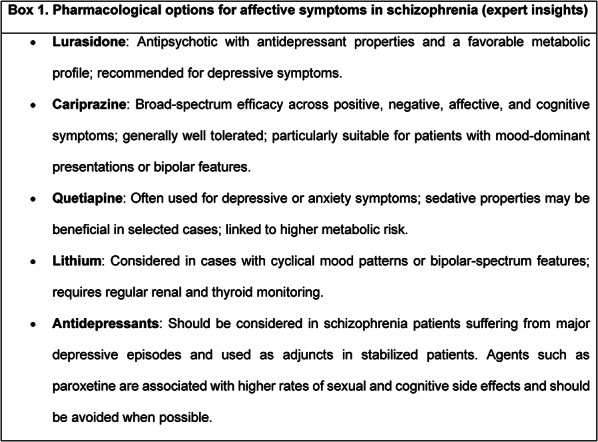


### Sexual dysfunction in schizophrenia

#### Sexual dysfunction: clinical challenges and real-world reflections

Sexual dysfunction is a common but underrecognized issue in schizophrenia, frequently linked to antipsychotic or adjunctive treatments. Many patients are reluctant to disclose sexual concerns spontaneously; therefore, symptoms often remain undetected unless clinicians initiate the discussion. Given these barriers, a systematic assessment and an open, nonjudgmental dialogue are essential to comprehensive management [72].

Far from being a minor side effect, sexual dysfunction is a central clinical issue with direct consequences for treatment adherence, therapeutic relationships, emotional intimacy, and psychosocial functioning [17, 18]. Evidence confirms its strong association with reduced continuation of antipsychotic treatment [73].

Antipsychotic-induced sexual dysfunction has a complex neurobiological basis. Dopaminergic blockade can lead to hyperprolactinemia, which disrupts hormonal cascades regulating libido, orgasm, and reproductive function [74, 75]. These effects are not limited to adults: in youth, elevated prolactin has been associated with both sexual dysfunction and breast abnormalities [76]. Hyperprolactinemia occurs more frequently in females and, when prolonged, may cause menstrual irregularities, hypogonadism, infertility, osteoporosis, and may contribute to long-term endocrine complications. Although a potential association between hyperprolactinemia and breast cancer has been suggested, current evidence remains inconclusive. Moreover, several other factors, such as obesity, nulliparity, diabetes, smoking, and reduced breastfeeding, are likely to contribute more substantially to overall breast cancer risk in women with schizophrenia [77–79]. Beyond prolactin, serotonergic (5-HT2) and adrenergic (α1) mechanisms may also contribute to reduced desire, arousal, and orgasmic difficulties [80]. Importantly, sexual dysfunction and prolactin elevation have been observed even before antipsychotic exposure in first-episode psychosis [81], suggesting that intrinsic illness-related mechanisms may compound treatment-related risks. Amisulpride is among the strongest prolactin-elevating antipsychotics, even at low antidepressant-range doses and at higher antipsychotic doses. Despite its metabolically favorable profile compared with other second-generation antipsychotics, amisulpride should therefore be avoided in patients with existing or high-risk sexual dysfunction [82].

Clinical thresholds for hyperprolactinemia are defined as mild (25–50 ng/mL), moderate (50–100 ng/mL), and severe (>100 ng/mL), with vigilance warranted even at moderate levels [83]. Spanish consensus guidelines recommend routine prolactin monitoring, baseline bone density screening in high-risk groups, and early intervention in symptomatic cases [82]. Osteoporosis screening should be considered for all patients with sustained hyperprolactinemia, with risk assessment based on hormonal factors (e.g., estradiol, progesterone), age, and sex, rather than relying solely on overt symptoms such as amenorrhea and/or galactorrhea. This is particularly important for postmenopausal women and individuals under 25 years of age, in whom early bone density loss may have long-term health consequences.

Fertility and reproductive goals should be addressed proactively before starting prolactin-elevating antipsychotics, especially in younger women. Such conversations are essential for informed decision-making and long-term reproductive planning. However, cultural stigma and taboos surrounding sexuality, particularly around topics such as masturbation, sexual orientation, or intimacy, remain significant barriers to open communication.

Most psychiatrists lack formal training in assessing or managing sexual health, leaving them unprepared to address this prevalent and clinically relevant issue. A cross-sectional study found that 73% of psychiatrists do not routinely inquire about sexual side effects. In comparison, only 35% of patients report experiencing them, and even fewer disclose such effects without being directly asked [84]. Moralistic or paternalistic attitudes in psychiatric care can further discourage open discussion, leading to covert nonadherence, disengagement from treatment, and erosion of therapeutic trust.

The psychosocial consequences of untreated dysfunction are considerable, contributing to social withdrawal, relational instability, and, in some cases, engagement with sex workers, particularly among male patients [17]. While 60% of patients with psychosis express interest in sexual and emotional connections, only 13% maintain stable sexual relationships. Masturbation is common, and engagement with prostitution is nearly twice as frequent as in the general population [17, 85]. These unmet needs contribute to treatment discontinuation – affecting up to 36% in men with sexual dysfunction – and highlight the importance of structured assessment and integration of patient concerns into care [86].

Sexual dysfunction frequently co-occurs with other vulnerabilities, including cognitive impairment, emotional dysregulation, and increased risk of abuse or sexually transmitted infections. These risks may be especially pronounced in women, who are often more vulnerable due to combined cognitive and psychosocial impairments [87]. A structured, measurement-based assessment of sexual function prior to initiating antipsychotic treatment can facilitate open dialogue, differentiate pre-existing dysfunction from treatment-emergent effects, and guide medication choices.

Incorporating validated tools into routine care could improve symptom detection, monitoring, and treatment outcomes. The PRSexDQ-SALSEX is a brief, sensitive, and well-validated tool in both schizophrenia and depression, accepted by patients, and available in multiple languages [80, 88, 89], yet remains underused in practice. The lack of dedicated sexology services in many psychiatric systems, along with stigma or reluctance among other medical specialists (e.g., endocrinologists, gynecologists), contributes to fragmented care for patients with severe mental illness. Psychiatrists are therefore called upon to assume a central role in addressing sexual health as part of person-centered care.

A persistent challenge in this field is the scarcity of randomized controlled trials, as efforts to build a stronger evidence base are frequently hindered by low patient recruitment and limited clinician engagement. Even when sexual dysfunction is identified, care is often delayed by unclear referral pathways and poor access to specialist services, underscoring the need for structured tools, protocols, and evidence-based management strategies within psychiatric settings.

#### Sexual dysfunction: pharmacological strategies and key considerations

Switching antipsychotics is often an effective strategy, especially when transitioning from agents that induce hyperprolactinemia (e.g., risperidone or paliperidone) to alternatives associated with lower prolactin elevation, such as aripiprazole, cariprazine, quetiapine, or olanzapine. These agents can reduce sexual side effects while maintaining adequate psychiatric symptom control.

The management of antipsychotic-induced sexual dysfunction remains largely empirically guided. Common strategies include dose reduction, switching to prolactin-sparing agents (e.g., aripiprazole, quetiapine, ziprasidone), and the cautious use of adjunctive agents such as dopamine agonists [90]. However, supporting evidence is limited, and conducting randomized controlled trials in this area is challenging. The REMEDY pilot trial, designed to investigate antipsychotic switching, was discontinued early due to low recruitment, highlighting real-world barriers to both clinician and patient engagement with this topic [91, 92].

Adjunctive pharmacological treatments, such as phosphodiesterase inhibitors (e.g., sildenafil), have been shown to improve erectile dysfunction in patients with schizophrenia [93], yet they remain underused. This underuse is partly due to clinician discomfort, lack of training, and misconceptions about their role in psychiatric care. Targeted education and institutional support could help normalize their use where clinically appropriate.

Routine exploration of sexual health should be an integral part of psychiatric care. Prolactin and relevant sex hormone levels (e.g., testosterone, estrogen, progesterone) should be assessed in cases of sexual disturbances, symptoms suggestive of endocrine dysfunction, or other prolactin-associated complications. Particular consideration is warranted for younger patients and postmenopausal women, who may face increased hormonal vulnerability.

Evidence on newer agents, including aripiprazole, brexpiprazole, and cariprazine, suggests that prolactin-sparing properties may support sexual function in some patients. Emerging real-world data indicate that cariprazine may improve both motivational and sexual functioning, supported by a long half-life that facilitates adherence. A prospective Spanish study reported improvements in sexual function, hormonal profiles, and symptom stability [Montejo, data on file 2025].

Conversely, antipsychotics such as risperidone and paliperidone are strongly associated with hyperprolactinemia and its related complications, including sexual dysfunction and gynecomastia [17, 94–97]. When these agents are prescribed, close monitoring and early intervention are essential to mitigate adverse effects and maintain treatment engagement.

#### Sexual dysfunction: expert consensus with actionable recommendations

The panel developed seven consensus statements addressing the screening, prevention, and management of sexual dysfunction in individuals with schizophrenia. These recommendations underscore the importance of systematic assessment, targeted pharmacological strategies, interdisciplinary collaboration, and the routine integration of sexual health into psychiatric care. All seven statements reached consensus in Round 1, with three rated as very strong agreement and four rated as strong consensus (Table 2).

**Table 2:** Sexual dysfunction – Delphi consensus statements and level of agreement

No.	Statement	Agree or Strongly Agree	Median Score	Interpretation
1	Routine, structured assessment of sexual function should be embedded in standard psychiatric care. Validated tools such as the PRSexDQ-SALSEX should be used regularly to facilitate early detection of sexual dysfunction and promote open, clinically relevant dialogue	80%	6.5	Strong consensus
2	Clinicians must proactively initiate discussions on sexual health, as most patients do not disclose concerns unless directly asked	100%	7	Very strong agreement
3	Psychiatric training programs should include focused education on sexual health and psychotropic-induced sexual dysfunction to ensure core clinical competence in this area	100%	7	Very strong agreement
4	Institutions should implement protocol-based approaches to standardize the assessment and management of sexual dysfunction across services. Hospital-wide adaptations of internal clinical guidelines can enhance consistency and accountability	80%	6.5	Strong consensus
5	First-line antipsychotic selection should consider sexual side effect risk, especially in first-episode patients and those of reproductive age. Particular attention is warranted for women undergoing menopausal transitions, as hormonal changes may influence psychotic symptoms and require treatment adjustments	100%	7	Very strong agreement
6	An integrated care model is essential. Optimal management requires coordinated collaboration between psychiatry, primary care physicians, and specialties such as endocrinology, gynecology, urology, and sexual medicine	80%	6.5	Strong consensus
7	There is a pressing need for robust, well-designed clinical research focused on sexual health in schizophrenia. Priority areas include gender-specific outcomes, hormonal influences, reproductive concerns, and long-term treatment effects	80%	7	Strong consensus

Box 2 summarizes pharmacological options commonly considered for managing sexual dysfunction in schizophrenia, based on expert insights.


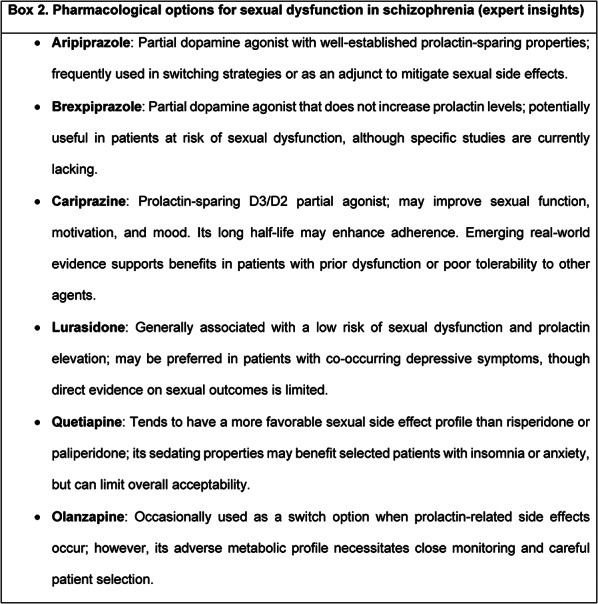


### Metabolic disturbances in schizophrenia

#### Metabolic disturbances: clinical challenges and real-world reflections

Metabolic disturbances, often precipitated or exacerbated by antipsychotic treatment, are a major driver of the excess cardiovascular morbidity and mortality in schizophrenia. Weight gain typically follows a nonlinear trajectory, with the most significant increases in the first 3 months, followed by a plateau. This pattern underscores the importance of initiating metabolic interventions early and raises concerns about their long-term effectiveness.

Emerging evidence suggests that such abnormalities may be present from illness onset, even before pharmacological exposure. Antipsychotic-naïve patients with first-episode psychosis have demonstrated elevated proinflammatory markers (e.g., IL-6 and TNF-α) that correlate with primary negative symptoms [98]. They have also shown higher fasting glucose, insulin resistance, triglyceride levels, and reduced high-density lipoprotein cholesterol compared with healthy controls [99–101]. These findings reinforce the need for risk monitoring and preventive strategies starting from the first clinical encounter. Notably, patients with lower baseline weight may be more susceptible to significant weight gain, challenging the assumption that metabolic risk is limited to those who are already overweight.

Genetic predisposition and baseline motivation have been identified as key factors influencing physical activity and lifestyle change. Some individuals may have an inherited tendency toward healthier habits, which can complicate the implementation of uniform lifestyle recommendations.

Genetic overlap between psychotic and metabolic disorders, particularly in first-degree relatives, offers an opportunity for early screening and more personalized prevention strategies.

Adherence to exercise and lifestyle changes is often hindered by barriers such as family pressure, unrealistic expectations, and limited resources. Clinicians emphasized the importance of using supportive, nonjudgmental approaches, avoiding blame, and fostering collaborative planning to promote sustained engagement. A recent integrative review further highlights the psychological, social, and structural barriers to physical activity in schizophrenia and outlines intervention strategies to support sustained physical activity participation [102].

People with schizophrenia are less likely to receive guideline-based interventions for cardiovascular risk factors, and, even when such care is provided, evidence-based treatment is often lacking. Psychiatrists, who are often the sole point of medical contact, are uniquely positioned and ethically obliged to assume a leadership role in managing cardiometabolic risk.

Access to newer metabolic treatments, such as glucagon-like peptide 1 receptor agonists (GLP-1 RAs), remains limited. Key barriers include cost, prescribing restrictions, concerns about psychiatric side-effects [103], and fragmented coordination between psychiatric and primary care services. These challenges raise ethical concerns about treatment equity.

Metabolic effects of antipsychotics are particularly relevant in younger patients. A recent meta-analysis found that children and adolescents with neurodevelopmental and neuropsychiatric disorders are especially vulnerable to metabolic dysregulation, underscoring the importance of minimizing early complications to reduce long-term health risks [25].

Nonpharmacological interventions, including dietary counseling, smoking cessation, and physical activity, remain essential components of metabolic care. Regular physical activity reduces cardiovascular risk independently of weight loss. Although smoking cessation is a major modifiable cardiovascular risk factor, it remains inadequately addressed in this population due to the lack of tailored programs. As a result, its potential impact on public health remains limited.

Integrating psychiatry into mainstream preventive health policy is essential, with mental health treated as a central pillar rather than a peripheral concern.

#### Metabolic disturbances: pharmacological strategies and key considerations

Metformin remains the most widely used first-line pharmacological intervention for antipsychotic-induced weight gain (AIWG), associated with an average weight loss of approximately 3 kg compared with placebo [104]. When used prophylactically, metformin can achieve up to 4 kg of weight reduction [105]. Although off-label for prevention, its use is supported by evidence, and gradual titration is recommended to improve tolerability. Some clinicians support the preventive use, despite limited regulatory guidance.

Partial dopamine agonists such as aripiprazole, brexpiprazole, and cariprazine offer lower metabolic risk while maintaining antipsychotic efficacy. They are increasingly prioritized in first-episode psychosis and may be used either as replacement therapies or as adjuncts in patients who have already experienced weight gain. Switching to these agents has been linked to the greatest weight reductions among pharmacological strategies for individuals with severe mental illness [106]. Conversely, clozapine and olanzapine carry the greatest metabolic burden, including substantial weight gain and elevations in glucose, cholesterol, and triglyceride levels. In contrast, agents such as lurasidone and ziprasidone demonstrate more favorable metabolic profiles, with some showing improvements over placebo, including reduced glucose levels (lurasidone), lower low-density lipoprotein cholesterol (cariprazine), and increased high-density lipoprotein cholesterol (aripiprazole/brexpiprazole) [23].

GLP-1 RAs (e.g., semaglutide) are effective second-line options for patients unresponsive to metformin. In one case series, switching to semaglutide led to an average weight loss of 8.67 kg over 12 months [107]. In clozapine-treated patients, a randomized controlled trial reported a 13.88% body weight reduction at week 36 compared with 0.42% in the placebo group [108], confirming earlier findings in patients treated with clozapine or olanzapine [109]. Clinicians have raised concerns about a potential risk of severe constipation when GLP-1 RAs are combined with highly anticholinergic agents such as clozapine, although this has not yet been substantiated in clinical trials [108]. A large-scale study involving over 107,000 individuals with overweight, obesity, or diabetes found no increase in psychiatric adverse events among those treated with GLP-1 RAs. In fact, improvements in mental health-related quality of life have been reported [103, 110]. However, most of these data have been derived predominantly from individuals without a psychiatric diagnosis, underscoring the need for further research to assess safety and efficacy in people with severe mental illness.

Emerging evidence also suggests that GLP-1 RAs may influence substance use behaviors, such as smoking and alcohol use, likely through modulation of reward pathways. Although not yet extensively studied in psychiatric populations, these findings broaden the potential therapeutic value of GLP-1 RAs [111, 112]. Another clinical consideration is the possible association between increases in body mass index and clinical improvement, particularly with potent agents such as clozapine and olanzapine. This has prompted debate about how best to balance efficacy with metabolic burden in treatment-resistant patients [113].

Topiramate, once considered for weight control, is now largely discouraged due to poor tolerability and cognitive side effects, especially when combined with anticholinergics. Tailored antipsychotic combinations, guided by a clear pharmacological rationale, may be preferable to empiric or ad hoc prescribing. For example, the combination of aripiprazole with clozapine, targeting distinct dopamine receptors, was associated with the lowest rehospitalization risk in a large observational study [114]. Fixed-dose combinations, used successfully in cardiology and HIV care, may help simplify complex regimens and improve adherence in patients requiring multiple psychotropic and metabolic agents.

Digital decision-support tools, such as Psymatik, can further personalize antipsychotic selection by integrating patient preferences and side-effect profiles across 32 antipsychotics and 37 antidepressants. Using the TOPSIS method (Technique for Order of Preference by Similarity to Ideal Situation), Psymatik ranks medications based on user-defined priorities and displays results using intuitive heatmaps, supporting shared decision-making, adherence, and long-term treatment success [24].

#### Metabolic disturbances: expert consensus with actionable recommendations

The panel developed seven consensus statements addressing the prevention, early detection, and management of metabolic disturbances in schizophrenia. These recommendations highlight the importance of early intervention, the routine incorporation of metabolic monitoring into psychiatric care, and the combined use of evidence-based pharmacological and nonpharmacological strategies. The panel also recognized the potential role of digital tools to support adherence and long-term outcomes. All seven statements reached consensus in Round 1, with five rated as very strong agreement and two rated as strong consensus (Table 3). An additional statement, regarding the preventive use of metformin in patients prescribed metabolically active antipsychotics, was introduced after Round 1 but did not meet the ≥ 75% consensus threshold in Round 2 (60% agreement) and was therefore classified as considered but not formally endorsed. Panel feedback reflected a range of perspectives: some supported the use of metformin when tailored to individual metabolic profiles and medical conditions, while others favored alternative approaches, such as GLP-1 RAs, pointed to variability in weight gain trajectories, or raised concerns about adherence and the lack of regulatory approval for preventive use.

**Table 3:** Metabolic disturbances: Delphi consensus statements and level of agreement

No.	Statement	Agree or Strongly Agree	Median Score	Interpretation
1	Metabolic monitoring and intervention should begin at the first clinical encounter, ideally when initiating antipsychotic treatment. Delaying action until significant weight gain occurs is not clinically acceptable	100%	7	Very strong agreement
2	Measurement-based, patient-centered care improves engagement and long-term outcomes. Risk stratification, patient preferences, and ongoing monitoring should guide personalized treatment strategies	100%	7	Very strong agreement
3	Routine metabolic screening must be integrated into psychiatric workflows, rather than considered optional or delegated to other specialties	100%	7	Very strong agreement
4	GLP-1 RAs show promising efficacy in managing AIWG and improving cardiometabolic outcomes. More evidence is needed to establish long-term psychiatric safety in people with serious mental illness	100%	7	Very strong agreement
5	Populations with neurodevelopmental disorders or intellectual disabilities require tailored metabolic strategies. Their increased vulnerability necessitates early intervention and carefully individualized prescribing	80%	7	Strong consensus
6	Psychiatric practice must evolve to include advanced pharmacological understanding, proactive metabolic management, and appropriate use of evidence-based combination strategies	100%	7	Very strong agreement
7	Digital tools such as Psymatik can support shared decision-making by aligning patient priorities with treatment profiles and enhancing adherence	80%	6.5	Strong consensus
8*	The preventive use of metformin should be considered when prescribing metabolically active antipsychotics, such as clozapine, to help attenuate weight gain	60%	6	Considered, but no formal consensus

*Statement introduced after Round 1. Qualitative feedback indicated that the use of metformin should be individualized, with alternatives such as GLP-1 RAs considered. Additional comments highlighted variability in weight gain patterns, as well as concerns about adherence and lack of regulatory approval for preventive use

Box 3 summarizes pharmacological options commonly considered for managing metabolic disturbances in schizophrenia, based on expert insights.


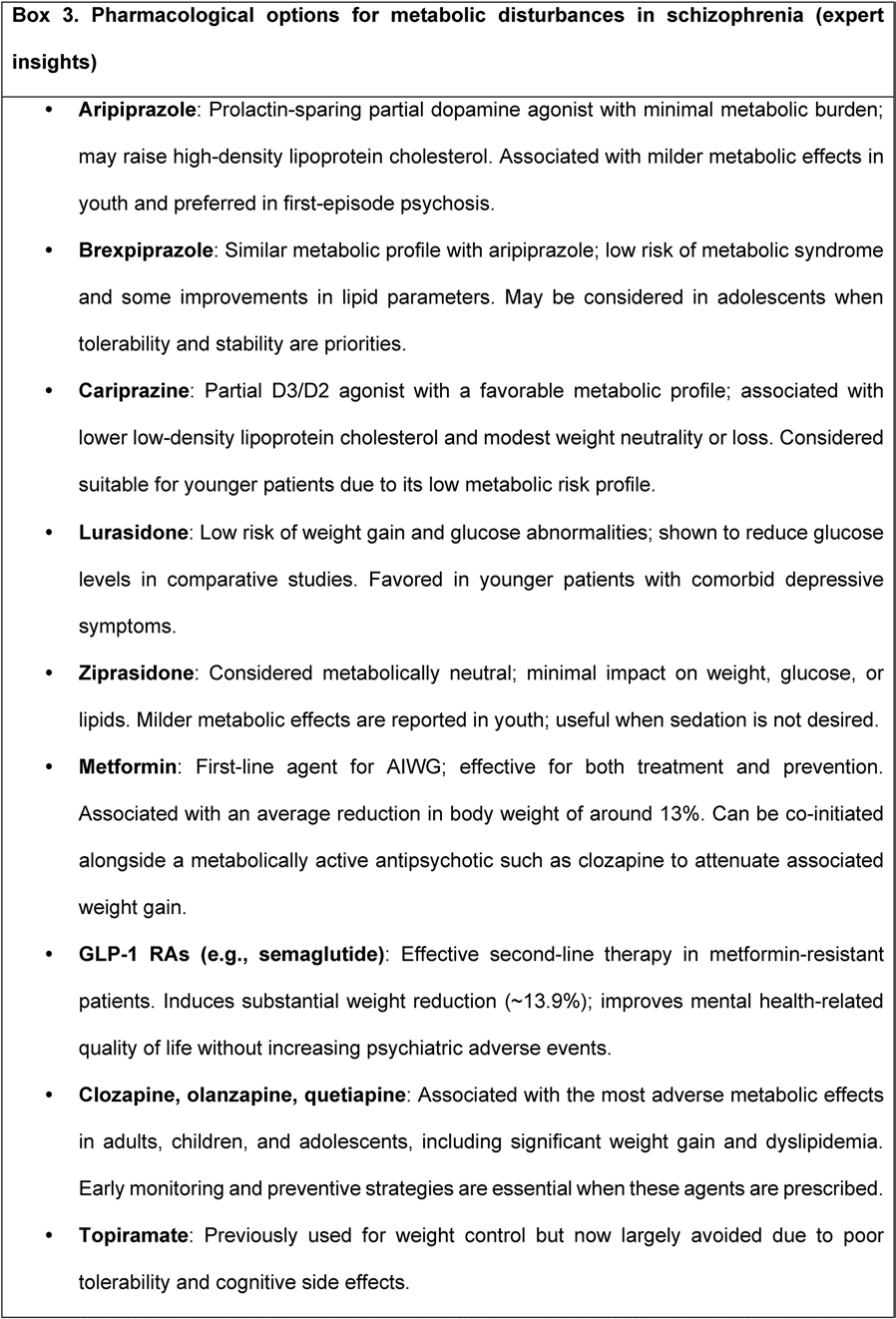


### SUD in schizophrenia

#### SUD: clinical challenges and real-world reflections

Cannabis use is highly prevalent among individuals with schizophrenia and, in the general population, has been associated with an increased risk of developing the disorder (odds ratio 3–4) [115]. The rising potency of modern cannabis – often exceeding 12% and, in some cases, reaching 35–40% tetrahydrocannabinol (THC) compared with historical averages of 2–4% – along with the emergence of synthetic cannabinoids, further amplifies this risk. These factors contribute to earlier onset and more severe psychosis, complicating dual diagnosis (also referred to as co-occurring disorder) management [116, 117]. Cannabis users with schizophrenia also tend to have poorer outcomes, including higher relapse rates and worse functional trajectories, underscoring the importance of early identification and intervention.

Tobacco use is another major concern [118]. Prevalence rates have been reported as high as 90% among individuals with schizophrenia in US population studies, compared with approximately 26% in the general population [33]. High rates of smoking are also observed internationally, though prevalence varies by country and population studied. Nicotine dependence remains particularly resistant to intervention, and many patients lack motivation to engage in cessation efforts, even when appropriate addiction services are available.

Transitions from child and adolescent to adult psychiatry services are high-risk periods, often marked by care discontinuity, reduced engagement, and increased relapse. Inconsistent service availability and coordination across regions exacerbate these vulnerabilities, limiting early intervention opportunities.

Sex-specific vulnerabilities also shape outcomes. Women with substance-induced psychosis are more likely to transition to bipolar disorder, while in men, cannabis- or multiple-substance-induced psychosis is often associated with a higher risk of progression to schizophrenia spectrum disorders [119]. These distinctions highlight the need for systematic monitoring and tailored care pathways.

Sustaining engagement with patients with dual diagnosis is challenging. Low motivation, cognitive impairment, emotional blunting, and chaotic lifestyles often require assertive outreach, long-term planning, and structured community-based support. Patients with chronic dual diagnosis often fall between service thresholds, remaining functionally impaired but no longer acutely psychotic. The absence of structured rehabilitation pathways for this population leads to fragmented and insufficient care.

An integrative approach is essential – not necessarily requiring dedicated “dual diagnosis” services, but ensuring that all psychiatric and addiction care settings implement coordinated strategies tailored to the individual and their evolving needs.

The early phase of schizophrenia presents a critical window for altering long-term outcomes. Decisions made in the first few years of illness significantly influence future trajectories, underscoring the importance of timely, comprehensive, and individualized intervention.

#### SUD: pharmacological strategies and key considerations

Long-acting injectable (LAI) antipsychotics, particularly aripiprazole, risperidone, and paliperidone, remain underused in patients with comorbid SUD, despite strong evidence supporting their effectiveness in improving adherence and reducing relapse risk [120]. This underuse represents a missed opportunity, especially for individuals with irregular routines, limited insight, and poor engagement. A recent multi-country interview study involving patients, caregivers, and prescribers reported favorable views toward a hypothetical 2-month LAI, highlighting reduced treatment burden and enhanced autonomy as key benefits [121].

Partial D2/3 agonists may also be considered in patients with schizophrenia and comorbid affective symptoms or cannabis use [122, 123]. More broadly, emerging evidence suggests that this class can improve outcomes in schizophrenia with comorbid cannabis use disorder, offering benefits for both psychotic symptoms and substance cravings [123]. Within this drug group, D3-preferring agents with favorable tolerability profiles and growing real-world evidence (e.g., cariprazine) may help address motivational deficits and substance-related behaviors [124]. Clozapine remains the most effective treatment for refractory schizophrenia [125] and may also benefit patients with persistent comorbid substance use [117, 126]. Nevertheless, its use is limited by monitoring demands, side-effect burden, and clinician reluctance, despite evidence of reduced suicidality [127].

Pharmacological regimens should be actively reviewed at key care transitions, especially at hospital discharge. While acute stabilization may require specific medications, discharge planning should align with long-term tolerability and adherence goals in community settings. Adjunctive therapies, such as transcranial magnetic stimulation (TMS) and GLP-1 RAs, are being investigated as potential interventions in comorbid SUD [128, 129], but evidence remains very limited. Most TMS studies in this setting have targeted nicotine dependence. Only one small trial addressed cannabis use alongside cognitive outcomes, without observing any clear effect. Research on GLP-1 RAs in SUD is also preliminary. Access to both interventions is constrained in many regions due to infrastructure, ethical/regulatory barriers, and reimbursement limitations.

Finally, tailored pharmacological combinations may be appropriate when targeting overlapping symptom domains. Clinicians emphasized the importance of using such combinations based on clear pharmacological rationale and aligning them with individual recovery goals, rather than relying on rigid monotherapy principles.

#### SUD: expert consensus with actionable recommendations

The panel developed seven consensus statements on the integration of psychiatric and addiction care for individuals with schizophrenia and comorbid SUD. These recommendations highlight the importance of coordinated service delivery, flexible diagnostic frameworks, patient-defined functional outcomes, and addressing systemic barriers to implementing best practices. Six statements reached consensus in Round 1, with four rated as very strong agreement and two rated as strong consensus. One additional statement, focused on systemic barriers to implementing best practices, was introduced after Round 1 and achieved very strong agreement in Round 2 (Table 4).

**Table 4:** Substance use disorders: Delphi consensus statements and level of agreement

No.	Statement	Agree or Strongly Agree	Median Score	Interpretation
1	Integrated care models are essential. The current fragmentation between psychiatric and addiction services undermines outcomes. Co-located teams, shared protocols, and cross-sector coordination are urgently needed.	90%	7	Very strong agreement
2	A dimensional, longitudinal diagnostic framework should replace rigid distinctions such as “primary” versus “substance-induced” psychosis. This enables more flexible and functional care planning.	80%	7	Strong consensus
3	Patient-defined functional outcomes, such as stable housing, relationships, and employment, should guide both pharmacological and psychosocial interventions.	90%	7	Very strong agreement
4	Nonmedical professionals play a pivotal role in engagement and continuity. Peer support workers, social networks, and community outreach should be fully integrated into core care delivery.	80%	6.5	Strong consensus
5*	System-level inertia, including fragmented care systems, rigid reimbursement policies, and compartmentalized funding mechanisms, remains a major barrier to implementing best practices. Addressing these obstacles is essential to promoting innovation and coordinated care.	90%	6	Very strong agreement
6	Funding gaps for long-term rehabilitation perpetuate fragmentation and restrict access. Mental health and general health systems must coordinate responsibilities to ensure sustained care delivery.	100%	7	Very strong agreement
7	SUD significantly increases suicide risk, not only in schizophrenia but also in bipolar disorder. Treatment planning must prioritize safety alongside pharmacodynamic fit, tolerability, and long-term adherence.	100%	7	Very strong agreement

*Statement introduced after Round 1 and evaluated in Round 2

Box 4 summarizes pharmacological options commonly considered for managing SUD in schizophrenia, based on expert insights.


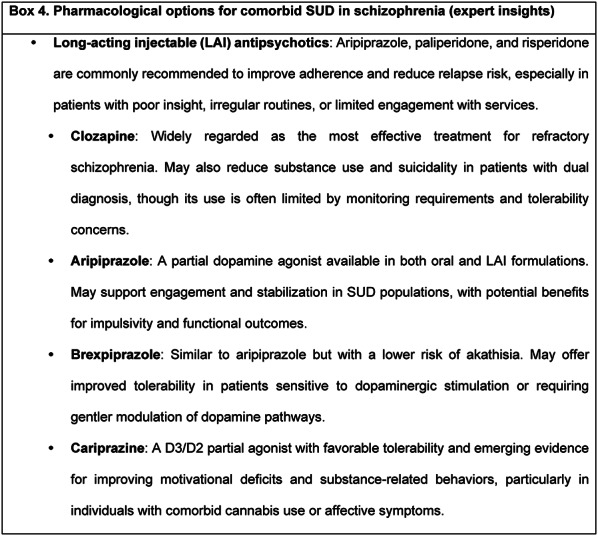


## Discussion

Schizophrenia is a multidimensional disorder frequently complicated by comorbid affective symptoms, sexual dysfunction, metabolic disturbances, and SUDs. These comorbidities profoundly influence treatment selection, adherence, recovery trajectories, and mortality, yet remain underrecognized and inconsistently addressed in routine practice [9, 14, 15].

Affective symptoms, particularly depression and anxiety, are highly prevalent but frequently underdiagnosed because of overlapping presentations, diagnostic ambiguity, and the lack of reliable biomarkers [16, 130]. These symptoms are strongly associated with functional decline, elevated suicide risk, and worsened physical health outcomes [1].

Antipsychotics with efficacy across affective and cognitive domains are increasingly favored for their broad-spectrum activity and favorable tolerability profiles [66, 69, 131, 132]. When adjunctive antidepressants are required, it is important to note that SSRIs, particularly paroxetine, are associated with a higher risk of sexual and metabolic side effects, whereas SNRIs may be preferred when these risks must be minimized [72, 133].

Sexual dysfunction remains a prevalent and clinically significant concern in schizophrenia, frequently linked to antipsychotic-induced hyperprolactinemia or sedative burden. Switching to a prolactin-sparing antipsychotic or cautiously introducing adjunctive strategies such as PDE5 inhibitors (only for erectile dysfunction) provides therapeutic options; however, randomized trial evidence remains limited [90, 92, 134]. Greater attention to sex-specific impacts and reproductive concerns in schizophrenia is needed [135]. Addressing these issues requires coordinated assessment and collaboration across relevant specialties [72, 82, 86].

Metabolic disturbances are a leading cause of the premature mortality observed in schizophrenia and may predate antipsychotic initiation [12, 24]. Despite increasing awareness, early intervention is hampered by siloed care and inconsistent screening. Partial dopamine agonists have more favorable metabolic profiles and should be considered early, especially in first-episode psychosis [26, 60, 136]. Adjunctive pharmacological strategies (e.g., metformin or GLP-1 RAs) also support weight and glucose regulation [105, 106]. However, access to GLP-1 RAs is often constrained by cost, reimbursement restrictions, and prescribing regulations, which limit their availability in routine clinical practice despite strong evidence for metabolic benefit. Digital tools, such as Psymatik, can further support personalized treatment and shared decision-making [24, 26].

SUDs are highly prevalent in schizophrenia and exacerbate psychiatric symptoms, impair insight, and increase the risk of relapse, hospitalization, and suicide [32–34]. Rising THC concentrations in cannabis contribute to earlier onset and more severe psychosis [116]. Integrated dual diagnosis care remains uncommon but is urgently needed [117, 137]. Bridging psychiatry and addiction care through co-located teams, shared protocols, and dimensional diagnostic models is essential [138]. Several European regions already provide established examples of this approach. In the UK, integrated dual-diagnosis care is supported nationally through NICE guidance and implemented regionally through services such as the West Sussex dual diagnosis program and Greater Manchester Mental Health NHS Foundation Trust [139–141]. In Spain, regional structures such as Madrid Salud’s *Unidad de Patología Dual*, the Addictive Behaviours Unit at Hospital Clínic Barcelona, and specialized pathways at Fundació Hospitalària Sant Boi provide co-located mental-health and addiction services [142–144]. In Italy, regional networks such as Lombardy’s *Dipartimento di Salute Mentale e Dipendenze* integrate psychiatric and addiction care under a unified governance model, enabling coordinated multidisciplinary management [145]. Collectively, these examples demonstrate that integrated dual diagnosis pathways are feasible and already operational within European mental-health systems.

**Fig. 1:**
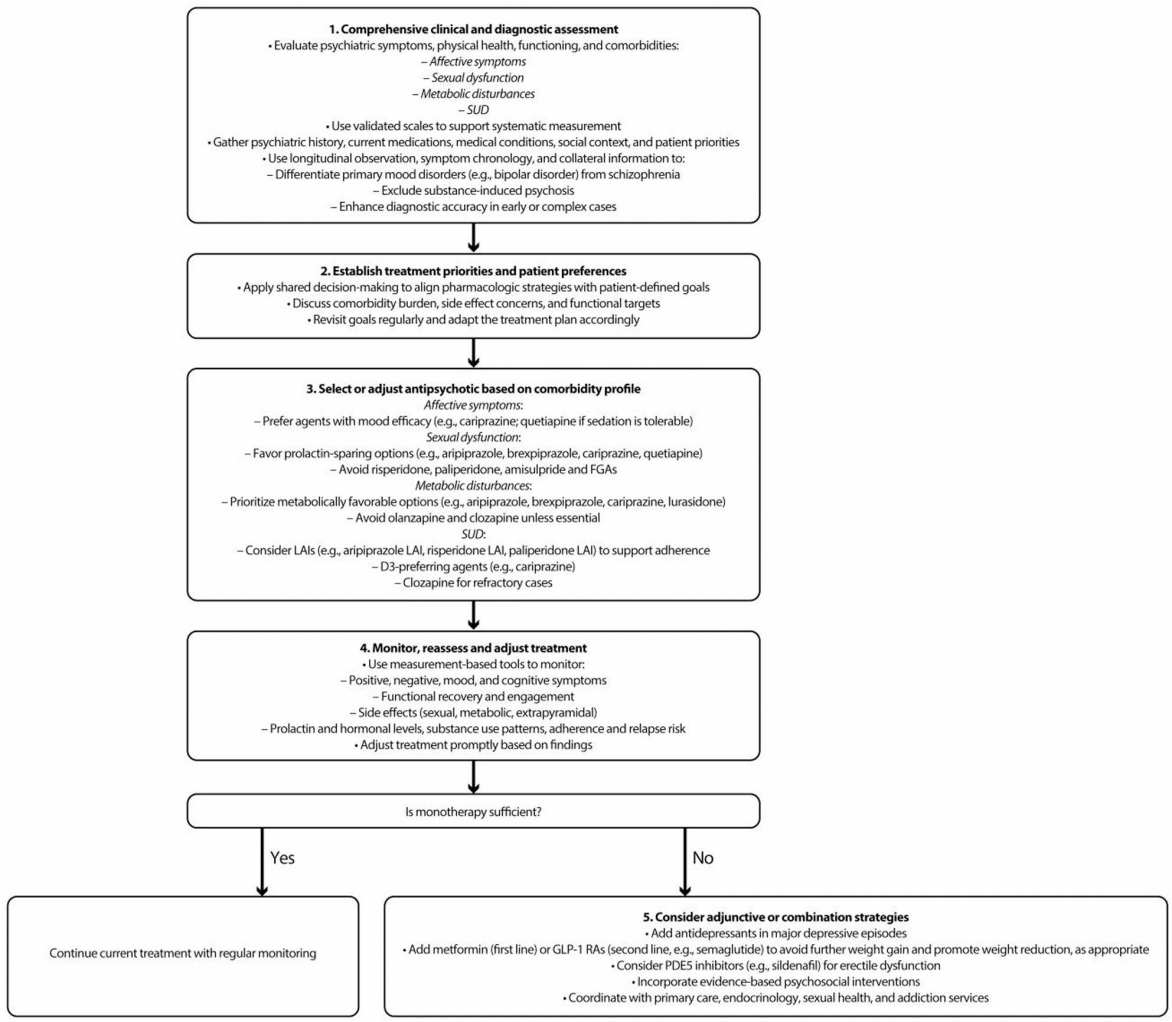
Pharmacological decision-making algorithm in schizophrenia with comorbidities

Certain partial dopamine agonists may offer both antipsychotic and anticraving effects and are increasingly considered in patients with dual diagnosis, i.e., the comorbidity of SUD with schizophrenia [53]. Real-world data, including a 6-month observational study in patients with comorbid cannabis use, support potential benefits in dual diagnosis populations [146].

Across symptom domains, the three available partial dopamine agonists (i.e., aripiprazole, brexpiprazole, and cariprazine) offer favorable tolerability, with lower risks of weight gain, prolactin elevation, and sedation [147]. Each agent has distinct pharmacological features that may inform selection based on comorbidity and patient priorities.

Aripiprazole is widely used and generally well tolerated, although activating side effects such as akathisia may occur in some patients [148, 149]. Its availability in both oral and LAI formulations supports engagement in patients with adherence difficulties, including those with comorbid SUD [150].

Brexpiprazole offers a more balanced receptor profile and lower risk of extrapyramidal symptoms, making it suitable for individuals sensitive to dopaminergic stimulation [66, 151, 152]. It is also prolactin-sparing and metabolically favorable, supporting its use in patients with sexual or metabolic vulnerability.

Preclinical and translational evidence highlights the therapeutic relevance of D3 receptor engagement for affective and negative symptom domains [153–155]. Cariprazine, a D3-preferring partial agonist, has demonstrated efficacy in primary negative symptoms [156–158], cognition [159], and mood symptoms, including antidepressant effects [2]. Its high affinity for D3 receptors, implicated in mood regulation, motivation, reward processing, and cognition [160], has been demonstrated in vitro and in vivo [161–165] and may contribute to these therapeutic effects [132]. Cariprazine is effective in schizophrenia and bipolar disorder and is increasingly considered a first-line option for patients with significant mood disturbances or bipolar features [122, 166]. Additional evidence supports benefits in patients with bipolar features [166–168], motivational impairments [67, 69, 132, 136, 169–171], and comorbid SUD [53]. Preclinical studies also suggest reductions in cocaine reward and relapse [52], and clinical and observational studies report improvements in symptom severity and reductions in cannabis, alcohol, and stimulant use [146, 171–174]. While encouraging, these findings require confirmation in larger randomized controlled trials.

Although partial dopamine agonists are included in several national and international guidelines for treating schizophrenia, their specific role in managing comorbid affective symptoms, metabolic disturbances, or SUD remains underdeveloped [26, 175, 176]. This expert consensus expands existing recommendations by contextualizing the use of these agents within real-world, comorbidity-informed strategies.

Rational combination strategies may be required when monotherapy is insufficient, particularly in patients with complex or refractory presentations [67, 177, 178]. Examples include combining antipsychotics with complementary receptor profiles (e.g., cariprazine + clozapine) [179–181], or adding adjunctive agents such as metformin, GLP-1 RAs, antidepressants, or mood stabilizers [103, 105, 182, 183]. These decisions should be based on pharmacodynamic rationale and patient-defined priorities.

Adherence remains a major challenge. Even brief treatment interruptions substantially increase the risk of relapse and hospitalization [184, 185]. In a recent study, nonadherence was especially prevalent among patients with comorbid SUD and severe psychopathology [186]. Side effects, including sexual dysfunction, weight gain, and sedation, continue to undermine long-term engagement [187]. Addressing these barriers requires tolerability-conscious prescribing, early side-effect management, and proactive use of LAIs where appropriate [26, 40, 117].

System-level obstacles, including fragmented care, underdiagnosis of comorbidities, and lack of interdisciplinary collaboration, limit the translation of evidence into routine practice. The expert panel emphasized the urgent need for integrated models, co-located services, interdisciplinary education, and broader adoption of measurement-based tools. Tables 5 and 6 provide a consolidated overview of the clinical challenges identified and the corresponding pharmacological and adjunctive strategies derived from the consensus process.

As with any consensus-based initiative, several limitations should be acknowledged. This work reflects expert consensus rather than systematic meta-analytic evaluation; accordingly, the recommendations represent the synthesis of available literature and collective clinical experience of the panel. Although this initiative received an unrestricted educational grant, multiple safeguards were implemented to preserve methodological rigor and independence. The sponsor had no role in literature review, evidence interpretation, expert discussion, statement development, Delphi voting, or manuscript preparation. All scientific activities were conducted independently, and the structured, anonymous Delphi rounds further minimized dominance effects and supported independent judgment. The additional statement introduced after Round 1 did not reach the predefined consensus and is transparently reported as such.

Differences in the frequency of references to specific antipsychotics reflect the underlying distribution of published evidence across comorbidity domains rather than a preference for any specific drug. These recommendations are intended to support real-world clinical decision-making and should not be interpreted as prescriptive treatment standards.

Finally, we recognize the essential role of national and international guideline-producing bodies and scientific organizations in developing formal evidence-based recommendations. Expert consensus statements such as the present work are intended to complement, rather than replace, these guidelines, particularly in areas where comorbidity-informed guidance remains limited and individualized, clinically informed decision-making is required.

In conclusion, optimizing care for individuals with schizophrenia and comorbidities requires a shift toward comorbidity-informed, patient-centered pharmacological care. Early detection, precision in treatment selection, digital support tools, and coordinated multidisciplinary engagement are essential to achieving long-term recovery, health equity, and improved quality of life. To support the clinical implementation of these recommendations, Fig. 1 outlines a pharmacological decision-making algorithm that synthesizes expert consensus and facilitates individualized treatment planning in schizophrenia complicated by comorbidities.

**Table 5:** Clinical challenges and expert consensus recommendations for managing schizophrenia with comorbidities*

Clinical challenge	Consensus-based recommendation	Agree or Strongly Agree	Median Score	Interpretation
**Underdiagnosis of comorbidities (affective**,** metabolic**,** sexual**,** SUD)**	Implement routine, structured screening across all stages of care; utilize validated tools such as CDSS PHQ-9 for depressive symptoms, PRSexDQ-SALSEX for sexual dysfunction, ASSIST for substance abuse, and metabolic monitoring protocols (e.g., weight, body mass index, glucose, and lipids), to detect comorbid symptoms early and guide individualized treatment.	80%	6.5	Strong consensus
**Adherence and engagement barriers; fragmented or siloed care pathways**	Combine proactive engagement strategies (e.g., outreach, peer support, individualized psychoeducation) with integrated care models that connect psychiatry with primary care, endocrinology, addiction services, and psychosocial rehabilitation; assign clear coordination roles to improve continuity and treatment adherence.	90%	6	Very strong agreement
**Limited pharmacological guidance for comorbidity management**	Enhance pharmacological guidance by aligning it with real-world comorbidity profiles, building on existing schizophrenia guidelines and emerging expert literature; promote clinician training in comorbidity-informed antipsychotic selection through updated protocols.	90%	7	Very strong agreement
**Comorbidity-informed antipsychotic selection**	Choose antipsychotics based on specific comorbidity profiles (e.g., mood, metabolic, substance use); prioritize agents with favorable tolerability and functional outcomes; integrate with psychosocial interventions when appropriate.	100%	7	Very strong agreement
**Concerns around combining antipsychotics**	Support tailored antipsychotic combinations when monotherapy proves inadequate; ensure combinations are pharmacologically rational, backed by emerging evidence, and clearly aligned with treatment goals.	80%	6.5	Strong consensus
**Elevated suicide risk**	Provide close monitoring during early illness stages, particularly in individuals with comorbid substance use or affective symptoms; incorporate routine suicide risk assessments into standard care; consider clozapine for high-risk patients.	90%	7	Very strong agreement
**Discontinuity during transitions (e.g.**,** youth to adult services)**	Develop structured transition protocols that facilitate shared care and handovers; ensure continuity of clinical services and eligibility across age transitions.	100%	7	Very strong agreement
**Limited access to advanced or adjunctive therapies (e.g.**,** rTMS**,** GLP-1 RAs)**	Advocate for improved access and reimbursement; integrate evidence-based adjunctive therapies into standard clinical algorithms.	80%	7	Strong consensus
**Variable implementation across healthcare systems**	Promote adaptable application of consensus principles, tailored to local infrastructure, regulations, and available resources.	100%	7	Very strong agreement
**Stigma in complex comorbid presentations**	Address stigma through staff training, inclusive language, and person-centered service design; actively involve peer support workers and individuals with lived experience in the treatment planning process.	100%	7	Very strong agreement
**Hormonal and reproductive health risks from antipsychotic-induced hyperprolactinemia**	Introduce routine endocrine monitoring (e.g., prolactin, estradiol, testosterone); assess bone density in high-risk groups (e.g., postmenopausal women, individuals under the age of 25); proactively discuss fertility and reproductive goals.	90%	7	Very strong agreement
**Clinician discomfort and lack of training in addressing sexual dysfunction**	Integrate structured sexual health education into psychiatric training and continuing professional development; promote routine use of validated screening tools (e.g., PRSexDQ-SALSEX) to facilitate open, nonjudgmental patient dialogue.	80%	6.5	Strong consensus
**Limited access to specialized care for sexual dysfunction**	Improve access to multidisciplinary sexual health services by expanding referral networks and fostering collaboration with endocrinology, gynecology, and sexual medicine; advocate for dedicated care pathways within mental health systems.	80%	6	Strong consensus

*The consolidated clinical challenges and expert recommendations were validated through a two-round modified Delphi process. All 13 statements met the predefined consensus threshold in Round 1, with eight receiving very strong agreement and five achieving strong consensus

**Table 6:** Pharmacological and adjunctive strategies by comorbidity profile in schizophrenia*

Comorbidity	Preferred antipsychotic strategies (pharmacological)	Adjunctive/Supportive interventions (non-pharmacological or non-antipsychotic)
**Affective symptoms**	• **Cariprazine**,** aripiprazole**,** brexpiprazole** (mood efficacy) • **Quetiapine** (if sedation is tolerated)	• Antidepressants (cautiously selected) • Mood stabilizers (e.g., **lithium**)
**Sexual dysfunction**	**Prolactin-sparing antipsychotics**: **• Aripiprazole**,** cariprazine**,** brexpiprazole**,** quetiapine** (low risk) **• Olanzapine** (moderate risk) **Avoid**: **• Risperidone**,** paliperidone**,** amisulpride**,** first-generation antipsychotics** (due to their strong prolactin-elevating potential)	• **PDE5 inhibitors** (e.g., sildenafil) for erectile dysfunction • **Adjunctive aripiprazole** to mitigate sexual side effects
**Metabolic disturbances**	• **Aripiprazole**,** cariprazine**,** brexpiprazole**,** lurasidone** (metabolically favorable) **Avoid**: • **Clozapine/olanzapine/quetiapine** unless clearly indicated	• **Metformin** (first-line for AIWG) • **GLP-1 RAs** (e.g., semaglutide)
**Substance use disorders**	• **Aripiprazole LAI; cariprazine; paliperidone LAI; risperidone LAI** • **Clozapine** (for treatment-resistant schizophrenia)	• **Consider LAI formulations** when adherence is unstable or during transitions in care

*This table presents expert-informed pharmacological strategies for managing schizophrenia complicated by affective symptoms, sexual dysfunction, metabolic disturbances, and substance use disorders. Adjunctive interventions should be tailored to symptom severity, individual tolerability, and delivered in coordination with relevant specialties. PDE5 inhibitors are included for their role in treating antipsychotic-induced erectile dysfunction

## Data Availability

The datasets generated during and/or analyzed during the current study are available from the corresponding author on reasonable request.

## Abbreviations

AIWG: Antipsychotic-induced weight gain
DSM: Diagnostic and Statistical Manual of Mental Disorders
EPS: Extrapyramidal side effects
GLP-1 RA: Glucagon-like peptide 1 receptor agonists
ICD: International Classification of Diseases
SUD: Substance use disorder
